# Arterial Stiffness is Associated with False-Positive ST-Segment Depression in Supine Bicycle Exercise Stress Echocardiography

**DOI:** 10.31083/j.rcm2402047

**Published:** 2023-02-06

**Authors:** Hyemoon Chung, Jiwon Seo, In Soo Kim, Jong-Youn Kim, Pil-Ki Min, Young Won Yoon, Byoung Kwon Lee, Bum-Kee Hong, Se-Joong Rim, Hyuck Moon Kwon, Eui-Young Choi

**Affiliations:** ^1^Division of Cardiology, Department of Internal Medicine, Kyung Hee University School of Medicine, 02447 Seoul, Republic of Korea; ^2^Division of Cardiology, Cardiovascular Center, Gangnam Severance Hospital, Yonsei University College of Medicine, 06273 Seoul, Republic of Korea

**Keywords:** exercise test, ST depression, arterial stiffness, myocardial ischemia, hypertensive response

## Abstract

**Background:**

Although exercise stress electrocardiography (ECG) is a popular tool for detecting coronary artery disease (CAD), the induced ST-depression without coronary artery stenosis (FST) remains a challenge for accurate diagnosis. Exercise-induced ST depression is related to poor prognosis even in non-obstructive disease; however, its determinants have not been fully defined. We sought to investigate whether ventriculo-vascular interactional indexes such as arterial stiffness index, exercise hemodynamic parameters and echocardiographic left ventricular (LV) functional parameters were related to FST.

**Methods:**

In the current study, 609 participants who underwent both supine bicycle exercise echocardiography and brachial-ankle pulse wave velocity (baPWV) measurement without exercise-induced regional wall motion abnormalities (RWMA) were analyzed. Referral reasons for stress test were CAD detection or evaluation of patency of previous revascularization. Stepwise graded supine bicycle exercise was performed with simultaneous ECG recording and echocardiography after full conventional resting echocardiography. The FST was defined as newly developed >1 mm ST depression without RWMA during exercise.

**Results:**

The median age of the study participants was 65 (59.0–70.5) years, and 222 (37%) patients were women. Among them, 103 (17%) patients showed FST during the exercise or recovery phase. The prevalence of FST did not differ between sexes. Older age, higher pulmonary arterial systolic pressure (PASP), left atrial volume index, baPWV and ankle brachial index at rest and hypertensive response, higher heart rate and rate-pressure product at peak exercise were significantly associated with FST. In multivariate analysis, higher peak heart rate, PASP, and baPWV were independently related to FST.

**Conclusions:**

Stress-induced RWMA in addition to ECG should be evaluated to detect CAD in patients with higher baPWV and PASP. FST might be linked to subclinical myocardial ischemia through arterial stiffness and diastolic dysfunction.

## 1. Introduction

Although exercise stress electrocardiography (ECG) is widely used and recommended for initial diagnostic test to detect coronary artery disease (CAD), false-positive ST depression (FST) remains a challenge for precise diagnosis [1, 2, 3]. Despite the relatively high false positive rate of exercise ECG for diagnosing obstructive CAD, exercise ECG paradoxically has strong prognostic value for future cardiovascular events and all-cause mortality, even in asymptomatic individuals or patients with low pre-test probability of CAD [4, 5, 6]. Therefore, we need to reveal the potential mechanism of FST during exercise. In addition, the prevalence of FST and the exact determinants of FST in supine bicycle exercise in patients with risk factors of CAD or previous history of revascularization, have not been fully understood [7, 8, 9]. Previous studies suggested that women, microvascular dysfunction and combined left ventricular hypertrophy, coronary milking phenomenon were potentially related to FST [2, 9, 10]. However, they did not accurately prove the physiological mechanism by basic experimental studies [10]. In addition, we easily meet the cases with FST without above situations.

Cardiac afterload is a major determinant of myocardial ischemic threshold [11]. There is a stiffness gradient from distensible elastic proximal arteries to muscular distal arteries in normal conditions [11, 12]. However, in a stiff arterial tree, the speed of propagation of the arterial pulse through the aorta is increased, and the increased speed of the forward traveling wave (pulse wave velocity) implies an earlier reflection of backward traveling wave from the periphery [11, 12]. Therefore, systolic blood pressure increases and diastolic pressure for coronary perfusion decreases in stiff aorta. However, relationship between arterial stiffness or exercise induced afterload index and FST has not been previously investigated.

Therefore, in this study we aimed to evaluate actual prevalence and potential associates to FST in patients with risk factors of CAD or previous history of revascularization using supine bicycle exercise stress echocardiography. In addition, we sought to investigate whether ventriculo-vascular interactional indexes such as arterial stiffness index, exercise hemodynamic parameters and echocardiographic left ventricular (LV) functional parameters were related to FST in cases without baseline ST depression, hypertrophic cardiomyopathy or dynamic LV outflow tract obstructions.

## 2. Materials and Methods

###  2.1 Study Participants

**Fig. 1.**
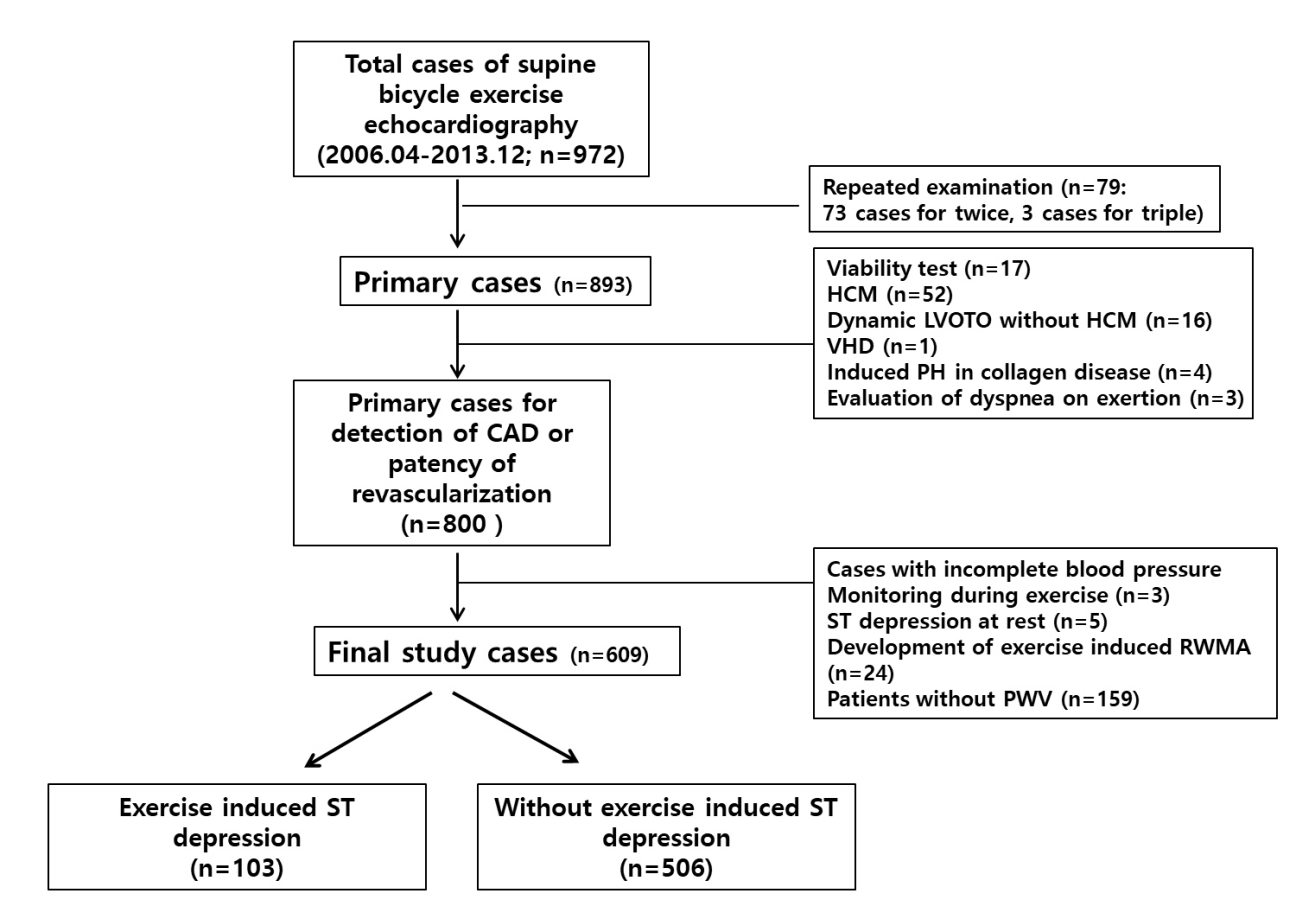
**Schematic illustration of study flow**. HCM, hypertrophic cardiomyopathy; LVOTO, left ventricular outflow tract obstruction; VHD, valvular heart disease; PH, pulmonary hypertension; CAD, coronary artery disease; FST, false ST-depression.

We retrospectively analyzed the results of supine bicycle exercise echocardiography from April 2006 to December 2013 at a single tertiary referral hospital. Referrals for an exercise echocardiography sought to detect CAD or to evaluate the patency of previous revascularization. Patients with concomitant cardiomyopathy, dynamic left ventricular (LV) outflow tract obstruction, valvular heart disease, or pulmonary artery disease were excluded. In addition, patients with baseline ST depression and exercise-induced regional wall motion abnormalities (RWMA) were also excluded. Finally, 768 patients were included in the study, of which 609 brachial-ankle pulse wave velocity (baPWV) measurements were performed within 1 month of exercise echocardiography (Fig. 1). Primary end points comprised horizontal or downsloping ST segment depression of ≥0.1 mV (1 mm), measured 80 ms after the J point, occurring in at least six consecutive complexes in at least three different leads (>1 mm) during exercise or recovery phase. The institutional review board of our hospital approved this study (2016-0378-001). The need for informed consents was waived due to the nature of the retrospective study.

### 2.2 Resting Echo-Doppler-Derived Hemodynamic Parameters

Before conventional echocardiography, blood pressure (BP) was measured on sitting position using an oscillometric blood pressure monitoring device (TM-2665P, AND, CA, USA). With echo-Doppler evaluation, LV mass index, relative wall thickness, LV ejection fraction, left atrial volume index (LAVI), mitral inflow pulse wave Doppler, and systolic and diastolic tissue velocities at the septal mitral annulus were measured. Pulmonary arterial systolic pressure (PASP) was calculated as 4 × ${\mathrm{(peak tricuspid regurgitant velocity)}}^{2}$ + right atrial pressure (RAP). Inferior vena cava diameter and its respiratory variation were assessed to measure RAP [13]. End-systolic pressure was calculated in the following formula; (2 × systolic BP + diastolic BP)/3. Stroke volume (SV) was calculated as 0.785 × ${\mathrm{(LV outflow tract diameter)}}^{2}$× (time velocity integral at LV outflow tract), and cardiac output (CO) was calculated as SV × heart rate. The total arterial compliance (TAC) [14] was calculated as SV/pulse pressure. The systemic vascular resistance (SVR) was calculated as 80 × (mean arterial pressure-RAP)/CO, and the effective arterial elastance (Ea) was estimated as the end-systolic pressure/SV [11, 14]. As used in previous studies, the ratio of mitral peak velocity of early filling to e’ (E/e’) was divided by the filling volume during diastole (SV) to estimate end-diastolic elastance (LV end-diastolic pressure/SV, end-diastolic elastance (Ed)) [15].

### 2.3 Measurement of Pulse Wave Velocity 

BaPWV were simultaneously measured using a vascular testing device (VP-2000; Colin Medical Technology, Komaki, Japan). After participants had rested in the supine position for >5 minutes, bilateral brachial and posterior tibial artery pressure waveforms were stored for 10 seconds by an extremity cuff connected to an oscillometric pressure sensor. Briefly, after an overnight fast and 5-min rest, the PWV was measured in the supine position. The electrocardiogram was monitored by electrodes on both wrists. Microphones placed at the left sternal edge in the third intercostal space were used to detect heart sounds. The baPWV, a marker for both central and peripheral arterial stiffness, was calculated using the equation (D1–D2)/T, where D1 is the distance between the heart and ankle, D2 is the distance between the heart and brachium, and T is the transit time between the brachial artery wave and tibial artery in the same side. The distance between the two sampling points was calculated based on the participant’s height, and the transit time was automatically determined from the time delay between the proximal and distal waveforms. The baPWV was calculated as the distance between the two arterial recording sites divided by the transit time. Ankle-brachial index (ABI) was calculated as the ratio of brachial and ankle systolic BP [11, 16].

### 2.4 Exercise Stress Echocardiography

Resting echocardiography images were obtained in the standard parasternal and apical views. Symptom limited multistage supine bicycle exercise testing was performed with a variable load bicycle ergometer (Model AE2, Medical Positioning, Inc., Kansas City, MO, USA). Patients pedalled at a constant speed starting at a workload of 25 watts (W), with the workload increasing by 25 W every 3 minutes according to ramp protocol. If patients could persist exercise, we did not stop the exercise. In cases who could not reach the 85% maximal heart rate, we did not apply the IV atropine. During exercise test, 12-lead ECG was simultaneously monitored. At each stage of exercise and recovery phase, 12-lead ECGs were printed out and recorded according to study protocol. Echocardiography was performed using a GE Vivid 7 ultrasound system (GE Medical systems, Horten, Norway) with a 2.5-MHz transducer during rest, exercise, and recovery. Patients who were taking beta blockers, we recommended to quit them from 3 days before exercise test. Pre-exercise baseline BP was measured in the supine position immediately before the exercise test. During exercise, the BP was measured at the end of each stage on the left arm using an oscillometric BP monitoring device (Tango, Tango+, SunTech medical, Morrisville, NC, USA). BP was measured after 1 minute, 3 minutes, and 5 minutes during recovery while stress images were acquired. At each stage of exercise and recovery, basal, mid-, and apical LV short axis views, along with apical 4-, 3-, and 2-chamber views, were obtained. Hypertensive response was defined as systolic BP ≥210 mmHg for men and ≥190 mmHg for women during exercise.

### 2.5 Statistical Analysis

The normality of distribution of continuous variables was assessed by Shapiro-Wilk test. Descriptions of continuous variables were presented as the mean ± standard deviation for variables with normal distribution and the median (interquartile range) for variables without normal distribution. Categorical variables were presented as number (%). Comparisons between FST and non-FST groups were analyzed by Student’s *t*-test for continuous variables with normal distribution and Mann-Whitney U test for variables without normal distribution. Chi-square analysis was performed for comparison of categorical variables. Logistic regression analysis (stepwise forward method) was performed to analyze the determinants of FST, and the covariates with *p* values < 0.05 in univariate analysis were included in the multivariate analysis. Two-sided *p* values < 0.05 were considered statistically significant.

## 3. Results

The median age of the study participants was 65 (59.0–70.5) years (64 in men vs. 66 in women, *p *< 0.001), and 222 (37%) were women. Among the study participants, 121 (20%) patients had a history of previous coronary revascularization, 169 (28%) had diabetes, and 140 (23%) took nitrate or nitrate-analogues. The patients’ median baPWV and ABI was 1497.0 (1345.5–1721.0) cm/s and 1.13 (1.08–1.18), respectively. The median exercise time and peak workload was 720.0 (540.0–900.0) sec and 100.0 (75.0–125.0) W, respectively. Their achieved maximal heart rate was 132 ± 17 bpm and 42% of the patients did not reach the 85% of age-predicted maximal heart rate. Among them, 103 (17%) patients showed FST during the exercise or recovery phases without induced RWMA. The incidence of FST did not differ between sexes (16.3% in men and 18.0% in women, *p* = 0.582). Comparisons of baseline and exercise echocardiographic characteristics are described in **Supplementary Table 1**. Patients with FST had older age, as well as higher LAVI, E/e’, and PASP (Tables 1,2). Regarding arterial stiffness index, both baPWV and ABI at rest were related to FST. The presence of hypertensive response, higher heart rate, and rate-pressure product at peak exercise were also significantly related to FST. In multivariate analysis, higher resting PASP and baPWV, in addition to peak heart rate at exercise, were independently related to FST (Table 3). Even after other parameters affecting baPWV, such as age, sex, and mean arterial pressure, were further included in the multivariable analysis, baPWV still remained significant for FST (OR 3.2 per m/s, 95% CI 1.608–6.460). In both sexes, baPWV was related to FST with comparable significance (OR 2.39 vs. 2.37), but it was not statistical significant due to the small number of population in women (in men OR 2.39 per m/s, 95% CI 1.035–5.506, *p* = 0.041; in women OR 2.37, 95% CI 0.945–5.932, *p* = 0.066).

**Table 1.** **Baseline characteristics**.

		Total	With FST	Without FST	*p* value
		(n = 609)	(n = 103)	(n = 506)	
Age, years		65 (59–71)	67 (61–73)	64 (58–70)	0.003
Males, n (%)		387 (66)	63 (61)	324 (64)	0.582
BSA, $m^{2}$		1.73 ± 0.17	1.70 ± 0.15	1.73 ± 0.17	0.072
Body mass index, kg/$m^{2}$		24.4 (23.0–16.5)	24.1 (22.8–26.5)	24.6 (23.0–26.5)	0.640
Hypertension, n (%)		466 (77)	79 (77)	387 (77)	0.962
Diabetes, n (%)		169 (28)	23 (22)	146 (29)	0.178
History of revascularization, n (%)		121 (20)	18 (18)	103 (20)	0.504
Resting SBP, mmHg		119 (107–132)	118 (107–132)	119 (107–132)	0.680
Resting DBP, mmHg		74 ± 11	72 ± 10	74 ± 11	0.061
Resting HR, bpm		67 (67–75)	67 (61–71)	68 (61–75)	0.204
Resting PP, mmHg		45 (36–54)	47 (37–56)	44 (35–53)	0.046
Medication					
	ARB or ACEi user, n (%)	540 (89)	93 (90)	447 (88)	0.569
	BB user, n (%)	173 (28)	35 (34)	138 (27)	0.169
	CCB user, n (%)	501 (82)	83 (81)	418 (83)	0.624
	Nitrate or its analogues user, n (%)	140 (23)	31 (30)	109 (22)	0.072
baPWV, cm/s		1497 (1346–1721)	1571 (1394–1848)	1485 (1341–1690)	0.012
ABI		1.13 (1.08–1.18)	1.14 (1.09–1.20)	1.13 (1.07–1.18)	0.032

FST, false-positive ST-depression; BSA, body surface area; SBP, systolic blood pressure; DBP, diastolic blood pressure; HR, heart rate; PP, pulse pressure; ARB, angiotensin receptor antagonist; ACEi, angiotensin converting enzyme inhibitor; BB, beta blocker; CCB, calcium channel blocker; baPWV, brachial-ankle pulse wave velocity; ABI, ankle brachial index.

**Table 2.** **Parameters of exercise echocardiography**.

	Total	With FST	Without FST	*p* value
	(n = 609)	(n = 103)	(n = 506)	
Peak SBP, mmHg	185 (168–202)	191 (168–203)	185 (167–201)	0.337
Peak DBP, mmHg	88 (81–97)	87 (80–97)	89 (82–97)	0.229
Peak HR, mmHg	133 (122–142)	136 (125–144)	133 (121–142)	0.149
Exercise time, sec	720 (540–900)	720 (540–900)	720 (540–900)	0.329
	(747.8 ± 220.6) *	(730.5 ± 207.6)	(751.3 ± 223.2)	(0.383)
Exercise capacity, watt	100 (75–125)	100 (75–125)	100 (75–125)	0.329
	(103.9 ± 30.6)	(101.5 ± 28.8)	(104.3 ± 31.0)	(0.383)
Peak workload, mmHg × bpm/1000	24.1 ± 5.0	24.9 ± 5.1	23.9 ± 4.9	0.024
Hypertensive response, n (%)	145 (24)	33 (32)	112 (22)	0.031
LVESP, mmHg	104.6 ± 14.2	104.4 ± 13.6	104.6 ± 14.3	0.884
SVR, dynes-sec-${\mathrm{cm}}^{–\,8}$	1.50 (1.25–1.74)	1.44 (1.24–1.69)	1.51 (1.25–1.74)	0.321
SV, mL	67.6 (57.8–78.0)	68.4 (60.6–79.8)	67.2 (57.0–77.1)	0.228
Ea, mmHg/mL	1.55 (1.31–1.83)	1.51 (1.28–1.77)	1.56 (1.32–1.85)	0.247
Ed	0.15 (0.12–0.20)	0.16 (0.12–0.20)	0.15 (0.12–0.19)	0.178
TAC, mL/mmHg	1.54 (1.21–1.89)	1.48 (1.18–1.85)	1.55 (1.21–1.90)	0.287
LVEDD, mm	45 (43–48)	45 (42–48)	45 (43–48)	0.766
LVESD, mm	30 (27–32)	30 (27–32)	30 (27–32)	0.986
RWT	0.42 (0.38–0.46)	0.43 (0.38–0.47)	0.42 (0.38–0.46)	0.117
LVMI, g/$m^{2}$	84 (74–97)	84 (74–96)	89 (78–101)	0.057
LAVI, mL/$m^{2}$	23.8 (20.0–27.6)	25.0 (21.4–29.6)	23.4 (19.5–37.2)	0.005
LVEF, %	66 (62–70)	66 (61–71)	66 (62–70)	0.828
e’, cm/sec	6.0 (5.0–7.0)	6.0 (5.0–7.0)	6.0 (5.0–7.0)	0.628
a’, cm/sec	9.1 (8.0–11.0)	9.0 (8.0–10.0)	9.8 (8.0–11.0)	0.255
s’, cm/sec	8.0 (7.0–9.0)	7.0 (7.0–9.0)	8.0 (7.0–9.0)	0.471
E/e’	10.3 (8.4–12.8)	10.6 (9.1–13.2)	10.2 (8.3–12.8)	0.043
PASP, mmHg	25 (22–28)	27 (23–30)	25 (22–28)	0.001

*mean ± standard deviation; FST, false-positive ST-depression; SBP, systolic blood pressure; DBP, diastolic blood pressure; HR, heart rate; LV, left ventricular; LVESP, LV end-systolic pressure; Ea, effective arterial elastance; Ed, end-diastolic elastance; TAC, total arterial compliance; SVR, systemic vascular resistance; SV, stroke volume; LVEDD, LV end-diastolic dimension; LVESD, LV end-systolic dimension; LVMI left ventricular mass index; RWT, relative wall thickness; LAVI, left atrial volume index; LVEF, LV ejection fraction; e’, peak early diastolic mitral annular velocity; a’ peak later diastolic mitral annular velocity; s’, peak systolic mitral annular velocity; E/e’, the ratio of mitral peak velocity of early filling to e’; PASP, pulmonary arterial systolic pressure.

**Table 3.** **Determinants of false positive ST-depression**.

	Univariate analysis		Multivariate analysis	
	OR (95% CI)	*p* value	OR (95% CI)	*p* value
Age, per year	1.04 (1.01–1.06)	0.008	1.02 (0.99–1.06)	0.160
Male	1.13 (0.73–1.75)	0.582		
Hypertension	1.01 (0.61–1.67)	0.962		
Diabetes	0.71 (0.43–1.17)	0.179		
ACEi/ARB use	1.23 (0.61–2.49)	0.570		
Calcium channel blocker use	0.87 (0.51–1.50)	0.624		
Beta-blockage use	1.37 (0.87–2.16)	0.170		
Nitrate use	1.57 (0.98–2.51)	0.061		
Systolic BP-resting, per mmHg	1.002 (0.99–1.01)	0.691		
Diastolic BP-resting, per mmHg	0.98 (0.962–1.001)	0.062		
Mean arterial pressure, per mmHg	0.99 (0.973–1.009)	0.338		
Pulse pressure, per bpm	1.01 (0.999–1.028)	0.062		
Resting heart rate, per bpm	0.98 (0.961–1.003)	0.090		
LV mass index, per g/$m^{2}$	1.01 (0.997–1.020)	0.129		
Relative wall thickness	6.92 (0.31–155)	0.222		
Exercise duration, per sec	1.000 (0.999–1.001)	0.383		
LVEF, per %	0.995 (0.968–1.024)	0.748		
s’, per cm/s	0.97 (0.86–1.08)	0.559		
e’, per cm/s	0.97 (0.86–1.09)	0.609		
E/e’	1.05 (0.998–1.107)	0.059		
LA volume index, per mL/$m^{2}$	1.03 (1.002–1.054)	0.037	1.02 (0.99–1.05)	0.225
PASP at rest, per mmHg	1.07 (1.02–1.11)	0.003	1.06 (1.02–1.11)	0.007
Peak heart rate, per bpm	1.014 (1.001–1.027)	0.039	1.02 (1.01–1.03)	0.007
Peak systolic BP, per mmHg	1.003 (0.993–1.013)	0.529		
Peak diastolic BP, per mmHg	0.99 (0.97–1.01)	0.231		
Hypertensive response at peak	1.66 (1.04–2.64)	0.033	0.96 (0.53–1.74)	0.881
Peak workload (rate-pressure product), per (mmHg × bpm)/1000	1.05 (1.01–1.10)	0.024		
baPWV, per m/s	2.40 (1.30–4.44)	0.005	2.75 (1.40–5.41)	0.003
ABI	16.33 (1.30–205.84)	0.031		

ARB, angiotensin receptor antagonist; ACEi, angiotensin converting enzyme inhibitor; BP, blood pressure; LV, left ventricular; LA, left atrial; LVEF, LV ejection fraction; e’, peak early diastolic mitral annular velocity; s’, peak systolic mitral annular velocity; E/e’, the ratio of mitral peak velocity of early filling to e’; PASP, pulmonary arterial systolic pressure; baPWV, brachial-ankle pulse wave velocity; ABI, ankle brachial index.

## 4. Discussion

### 4.1 Arterial Stiffness and Exercise-Induced ST Depression

Several previous studies showed that increased arterial stiffness is linked to endothelial dysfunction [17] in patients with CAD risk factors [18, 19, 20]. Although the aortic and peripheral arterial stiffness do not directly affect cardiac electrophysiology, they could affect LV diastolic function during aerobic exercise through ventricular-vascular interaction [21, 22]. In this study, although we did not measure the chamber diastolic function throughout the exercise test, increased baPWV could potentially affect electrical stability at peak exercise, which could then result in ST depression without definite RWMA. In addition, a previous study showed that arterial stiffness indexes, such as carotid-femoral PWV and carotid augmentation index were associated with reduced ischemic threshold in patients with moderate CAD [12]. Increased PWV shifts pressure wave reflections from diastole to systole reduce diastolic perfusion pressure [11]. Therefore, a stiff aorta has a diminished capacity to serve as a blood reservoir during cardiac ejection, such that blood is available for coronary perfusion during diastole [12].

### 4.2 Potential Mechanism of Exercise-Induced ST Depression 

The prevalence of FST was high in our study, which was consistent with previous studies [2, 9]. This might be due to the heterogeneous patient inclusion, which involved patients with a history of previous coronary revascularization, and supine bicycle exercise rather than upright exercise. The incidence of FST was also different according to exercise protocol. In the recent studies done by upright cycle ergometer exercise, the incidence of FST was 22% [2, 9] and 18.8% in treadmill exercise test [9]. The ST change could be a result of coronary microcirculatory dysfunction without radial contractile abnormality [2]. According to our study results, higher PWV, hypertensive response, and higher heart rate at peak exercise were independently correlated with FST. Relationship between higher PWV and FST reflects that resting afterload affects endocardial function through impaired vascular-ventricular interaction. In addition, relationship between hypertensive response to exercise and FST also reflects that exercise-induced increased afterload affects endocardial function through impaired vascular-ventricular interaction [22]. Some previous studies showed that hypertensive response was linked to FST without CAD [10, 23]. According to our study results, hypertensive response due to increased arterial stiffness [2, 24, 25, 26] is associated with FST, but hypertensive responses at a younger age can be linked to augmentation of SV due to vigorous LV contraction [24]. Therefore, in older adults with increased arterial stiffness, we need to take care of possibility of FST. In our study diastolic dysfunctional parameters, such as higher left atrial volume index and tricuspid regurgitant velocity, were linked to FST, indicating that ST depression without radial RWMA might not be a true “normal perfusion condition”, but rather an occult or a subclinical impaired perfusion status. This might support the poor prognosis of exercise-induced ST depression reported in several previous studies. When the ST segment downsloping is secondary to microvascular disease, the inducible subendocardial ischemia cannot achieve the critical mass to generate segmental wall motion anomalies of the LV. It was also reported that endothelial dysfunction could modify the repolarization process through the prolongation of repolarization phase at the subendocardial level [27]. Higher peak heart rate was related to FST possibly due to change in atrial repolarization according to previous study [28]. Contrary to some previous studies presenting higher incidence of FST in women [29, 30], the incidence of FST did not significantly differ between sexes in our study, despite higher trends in women. However, our study results were consistent with a recent large study performed in 3000 consecutive patients [9]. The study also showed an equal prevalence of FST in men and women, and concluded that FST in men could be predicted before the test with clinical characteristics such as left ventricular hypertrophy in ECG, known CAD and hypertension *etc*., while most cases in women could not [9]. A unique finding in our study was that baPWV also tends to be related to FST in women, which suggests that increased arterial stiffness may be a potential cause of FST in women. According to our study, higher heart rate and hypertensive response at peak exercise were linked to FST. This indicates that beta-blockers for heart rate reduction, nitrate for improvement in microcirculation, or antihypertensive medication can improve FST by reducing rate-pressure product. Some patients with exercise intolerance or those not trained for exercise may experience rapid heart rate elevation even during low intensity exercise [31, 32]. Therefore, graded and regular cardiopulmonary exercise training could be beneficial to prevent rapid heart rate elevation during exercise. Receptor for advanced glycation end-product antagonist, which potentially destiffen large arteries, may have favourable effects in preventing ST depression during exercise [33]. Nevertheless, further research is required.

### 4.3 Study Limitations

This study had several limitations. First, the patients included in the study were heterogeneous, from presenting symptoms of atypical chest pain to having a history of previous coronary revascularization. Therefore, small coronary vessel diseases or microcirculatory dysfunctional patients could be included. Second, although exercise-induced RWMA was thoroughly interpreted by both sonographers and cardiologists who were experts in echocardiography, by reviewing three short axis views and 4-, 3-, and 2 chamber views, the possibilities of missed RWMA, especially in the right coronary artery or left circumferential artery territory, still existed. However, as we reviewed the results one more time in patients with FST to detect missed RWMA, the possibility of missed RWMA was low. Third, in order to elucidate the potential mechanisms of ST change and its relationship with increased arterial stiffness at peak exercise, LV longitudinal systolic and diastolic function need to be evaluated. Fourth, as higher peak heart rate was linked to FST in our study, delta ST depression/delta heart rate, which has been shown to have higher sensitivity for CAD in a previous study [34], should be applied in future studies. Fifth, as we did not use intravenous atropine to achieve target heart rate due to symptom limited exercise test, some patients did not reach targeted maximal heart rate.

## 5. Conclusions

FST is not rare, especially in supine bicycle exercise. In cases with increased arterial stiffness, higher PASP and peak heart rate were related to exercise-induced ST depression. Therefore, stress-induced RWMA should be evaluated to detect epicardial coronary artery stenosis in patients with exercise-induced ST depression in ECG. Although not related to radial contraction abnormality, exercise induced ST depression without CAD might be associated with subclinical myocardial ischemia through arterial stiffness and subendocardial diastolic dysfunction.

## Data Availability

The datasets used and/or analyzed during the current study are available from the corresponding author on reasonable request.

## Supplementary Material

Supplementary material associated with this article can be found, in the online version, at https://doi.org/10.31083/j.rcm2402047.
