# Supplementary material for: Arterial Stiffness is Associated with False-Positive ST-Segment Depression in Supine Bicycle Exercise Stress Echocardiography
Source: Rev Cardiovasc Med. 2023 Feb 6;24(2):47. doi: 10.31083/j.rcm2402047 (PMC11273111; doi:10.31083/j.rcm2402047)
Supplement: Supplementary file 1 [file 2153-8174-24-2-047-s1.zip › 2153-8174-24-2-047-s1.docx]

**Supplementary Table 1.** Comparisons of baseline characteristics and parameters of exercise echocardiography between sex.

|  | **Total**  (n=609) | **Men**  (n=387) | **Women**  (n=222) | **P value** |
| --- | --- | --- | --- | --- |
| **Age**, years | 65.0 (59.0-70.5) | 64.0 (57.0-70.0) | 66.0 (60.0-72.0) | <0.001 |
| **FST**, n (%) | 103 (16.9) | 63 (16.3) | 40 (18.0) | 0.582 |
| **Body mass index**, kg/m^2^ | 24.4 (23.0-16.5) | 24.8 (23.3-26.5) | 24.6 (23.0-26.5) | 0.016 |
| **Hypertension**, n (%) | 466 (76.5) | 287 (74.2) | 179 (80.6) | 0.070 |
| **Diabetes**, n (%) | 169 (27.8) | 117(30.2) | 52 (23.4) | 0.071 |
| **History of revascularization**, n (%) | 121 (19.9) | 89 (23.0) | 32 (14.4) | 0.011 |
| **Resting SBP**, mmHg | 119.0 (107.0-132.0) | 118.0 (107.0-130.0) | 122.0 (107.8-134.3) | 0.110 |
| **Resting DBP**, mmHg | 73.9±10.9 | 73.9±10.9 | 73.8±11.0 | 0.812 |
| **Resting HR**, bpm | 67.0 (67.0-75.0) | 67.0 (61.0-75.0) | 67.5 (61.0-75.0) | 0.204 |
| **Resting PP**, mmHg | 45.0 (35.5-54) | 44.0 (35.0-52.0) | 46.0 (36.0-58.0) | 0.052 |
| **Medication** |  |  |  |  |
| **ARB or ACEi user**, n (%) | 540 (88.7) | 342 (98.) | 198 (89.2) | 0.759 |
| **BB user**, n (%) | 173 (28.4) | 120 (31.0) | 53 (23.9) | 0.060 |
| **CCB user**, n (%) | 501 (82.3) | 316 (81.7) | 185 (83.3) | 0.601 |
| **Nitrate or its analogues user**, n (%) | 140 (23.0) | 101 (26.1) | 39 (17.6) | 0.016 |
| **baPWV**, cm/s | 1497.0 (1345.5-1721.0) | 1474.0 (1331.0-1684.0) | 1546.0 (1374.0-1781.5) | 0.017 |
| **ABI** | 1.13 (1.08-1.18) | 1.13 (1.08-1.18) | 1.13 (1.08-1.19) | 0.894 |
| **Peak SBP**, mmHg | 185.0 (167.5-202.0) | 187.0 (168.0-205.0) | 181.5 (165.0-197.0) | 0.002 |
| **Peak DBP**, mmHg | 88.0 (81.0-97.0) | 88.0 (81.0-96.0) | 89.0 (81.8-97.0) | 0.285 |
| **Peak HR**, mmHg | 133.0 (122.0-142.0) | 131.0 (120.0-142.0) | 136.0 (126.0-144.0) | 0.010 |
| **Exercise time**, sec | 720.0 (540.0-900.0) | 900.0 (720.0-1080.0) | 540.0 (540.0-720.0) | <0.001 |
| **Exercise capacity**, watt | 100.0 (75.0-125.0) | 125.0 (100.0-150.0) | 75.0 (75.0-100.0) | <0.001 |
| **Peak workload**, mmHg*bpm/1000 | 24.1±5.0 | 24.1±4.8 | 23.9±5.29 | 0.592 |
| **Hypertensive response**, n (%) | 145 (23.8) | 67 (17.3) | 78 (35.1) | <0.001 |
| **LVESP**, mmHg | 104.6±14.2 | 104.1±13.8 | 105.5±14.8 | 0.244 |
| **SVR**, dynes-sec-cm^-8^ | 1.50 (1.25-1.74) | 1.48 (1.23-1.70) | 1.53 (1.33-1.83) | 0.042 |
| **SV**, ml | 67.6 (57.8-78.0) | 68.5 (59.5-79.8) | 65.5 (55.0-75.4) | 0.006 |
| **Ea**, mmHg/ml | 1.55 (1.31-1.83) | 1.51 (1.28-1.77) | 1.64 (1.36-1.93) | 0.001 |
| **Ed** | 0.15 (0.12-0.20) | 0.14 (0.11-0.18) | 0.17 (0.14 -0.22) | <0.001 |
| **TAC**, mL/mmHg | 1.54 (1.21 -1.89) | 1.48 (1.23-1.70) | 1.44 (1.09-1.85) | 0.002 |
| **RWT** | 0.42 (0.38 -0.46) | 0.42 (0.38-0.47) | 0.42 (0.38-0.46) | 0.938 |
| **LVMI**, g/m^2^ | 84.4 (74.0-97.0) | 87.1 (76.9-98.6) | 80.9 (71.0-92.7) | <0.001 |
| **LAVI**, mL/m^2^ | 23.8 (20.0 -27.6) | 23.5 (19.6-27.1) | 24.4 (19.9-28.4) | 0.117 |
| **LVEF**, % | 66.0 (62.0-70.0) | 66.0 (61.0-70.0) | 67.0 (63.8-71.0) | <0.001 |
| **e’**, cm/sec | 6.0 (5.0 -7.0) | 7.0 (5.0 -8.0) | 6.0 (5.0-7.0) | <0.001 |
| **a’**, cm/sec | 9.1 (8.0-11.0) | 10.0 (9.0-11.0) | 9.0 (8.0 -10.0) | 0.001 |
| **s’** , cm/sec | 8.0 (7.0-9.0) | 8.0 (7.0-9.0) | 7.0 (6.0-8.0) | <0.001 |
| **E/e’** | 10.3 (8.4-12.8) | 9.6 (8.1-11.8) | 11.4 (9.4-13.8) | <0.001 |
| **PASP**, mmHg | 25.0 (22.0-28.3) | 24.8 (22.0-28.0) | 25.4 (22.6-29.0) | 0.026 |

SBP, systolic blood pressure; DBP, diastolic blood pressure; HR, heart rate; PP, pulse pressure; ARB, angiotensin receptor antagonist; ACEi, angiotensin converting enzyme inhibitor; BB, beta blocker; CCB, calcium channel blocker; baPWV, brachial-ankle pulse wave velocity; ABI, ankle brachial index LV, left ventricular; LVESP, LV end-systolic pressure; Ea, effective arterial elastance; Ed, end-diastolic elastance; TAC, total arterial compliance; SVR, systemic vascular resistance; SV, stroke volume; LVEDD, LV end-diastolic dimension; LVESD, LV end-systolic dimension; LVMI left ventricular mass index; RWT, relative wall thickness; LAVI, left atrial volume index; LVEF, LV ejection fraction; e', peak early diastolic mitral annular velocity; a’ peak later diastolic mitral annular velocity; s', peak systolic mitral annular velocity; E/e’ the ratio of mitral peak velocity of early filling to e’; PASP, pulmonary arterial systolic pressure
